# Editorial: Novel biomarkers for potential clinical applications in lung cancer

**DOI:** 10.3389/fonc.2024.1481799

**Published:** 2024-09-13

**Authors:** Hongda Liu, Miao Liu

**Affiliations:** ^1^ Department of General Surgery, First Affiliated Hospital, Nanjing Medical University, Nanjing, China; ^2^ Department of Pathology, Brigham and Women’s Hospital and Harvard Medical School, Boston, MA, United States

**Keywords:** lung cancer, prognosis, biomarker, clinical application, signal pathway

Biomarkers play a critical role in modern medicine, significantly enhancing early diagnosis, prognostic evaluation, and personalized treatment across various diseases, including cardiovascular diseases, neurodegenerative disorders, infectious diseases, and cancers (1–4). Their application greatly improved diagnostic accuracy and increased opportunities for early intervention. In lung cancer, biomarkers such as EGFR, ALK, and ROS1 gene mutations were particularly important for guiding precision therapy and developing personalized treatment plans, resulting in significantly better clinical outcomes. The expanding use of biomarkers drove the evolution of medicine toward greater precision and personalization. The ongoing development of novel biomarkers for clinical applications in lung cancer further advanced this trend, improved treatment outcomes and survival rates, and made these biomarkers essential in lung cancer management.

Many biomarkers for various diseases were proven in numerous studies to be useful for predicting the prognosis of different diseases. Some studies have proved that the Systemic Immune-Inflammation Response Index (SIRI) reflected the prognostic significance in lung cancer patients undergoing microwave ablation (MWA). In a study involving 265 patients, the relationship between preoperative SIRI levels and long-term outcomes, such as overall survival (OS) and disease-free survival (DFS), was analyzed. The results indicated that higher SIRI levels were significantly associated with poorer long-term outcomes, including lower OS and DFS rates. Additionally, the study introduced nomograms based on SIRI and other independent factors to predict patient prognosis, demonstrating high accuracy. These findings highlighted the importance of SIRI as a prognostic tool, which might help clinicians identify high-risk patients and develop personalized treatment strategies for those undergoing MWA Wang et al. This also suggested the potential for cross-talk between signaling pathways across different diseases.

As new biomarkers continued to be identified, related biomarkers also emerged for some of the rarer types of non-small cell lung cancer (NSCLC). Recent research significantly enhanced our understanding of primary pulmonary lymphoepithelioma-like carcinoma (PPLELC), a rare subtype of NSCLC with distinct epidemiological and clinical features. Notably, PPLELC was more prevalent among Asian populations, a trend linked to the Epstein-Barr virus (EBV), which was uncommon in other lung cancer types. This viral connection suggested PPLELC might share etiological features with other EBV-related malignancies, such as nasopharyngeal carcinoma. Studies also shed light on the genetic landscape of PPLELC, identifying specific mutations, like those in the TP53 and CYLD genes, which correlated with distinct clinical outcomes. These genetic markers could help stratify patients into different risk categories, facilitating more personalized treatment approaches—a crucial development given the rarity of PPLELC and the lack of established treatment protocols. The identification of these biomarkers not only aided in accurate diagnosis but also paved the way for targeted therapies. Tailoring treatments to the genetic and molecular profile of PPLELC could significantly improve patient outcomes, particularly for those in advanced stages where conventional therapies fell short. Integrating these biomarkers into clinical practice would mark a significant advancement in PPLELC management, shifting from a one-size-fits-all model to more individualized strategies Zhang et al.

While most biomarkers were protein-based, non-protein biomarkers became increasingly important in the prognosis and treatment planning for NSCLC patients. Among these, circulating tumor DNA (ctDNA) showed great potential as a biomarker for predicting disease recurrence in patients with resectable stage I NSCLC. ctDNA was proven to serve as a non-invasive tool to identify patients at high risk of relapse, which was crucial for early intervention and monitoring. However, the study also indicated that ctDNA’s utility in guiding adjuvant therapy (ADT) decisions was limited, particularly in early-stage lung cancer. This suggested that while ctDNA held promise as a prognostic biomarker, further research and development were needed to improve its predictive accuracy and applicability in clinical decision-making, especially for treatment planning. The study contributed to the broader field of biomarker research by highlighting both the potential and the current limitations of ctDNA in lung cancer management, paving the way for more refined and effective use of biomarkers in the future Wang et al.

For predicting outcomes in lung cancer patients, various clinical and pathological factors were indispensable. In patients with resected stage I acinar- or papillary-predominant lung adenocarcinoma, the presence of non-predominant components, particularly micropapillary and solid components, was associated with poorer disease-free survival (DFS). When patients were categorized into risk subgroups based on these minor components, those with these high-grade patterns showed significantly worse prognoses. Conversely, the presence of lepidic components was associated with better outcomes Liu et al. Additionally, lymphovascular invasion, elevated carcinoembryonic antigen (CEA) levels, and a high platelet-to-lymphocyte ratio (PLR) were independent predictors of poor DFS. Comprehensive evaluation of these factors in the clinical management of early-stage lung adenocarcinoma was crucial, as they played a significant role in patient stratification and in guiding postoperative treatment strategies Bo Liu et al.

Some signaling pathways or mechanisms previously identified in tumors were also found to have new roles in lung cancer. A study, by analyzing tissue samples from patients, examined the expression of key proteins such as E-cadherin, beta-catenin, WNT proteins, and ECM components (such as chondroitin sulfate and various types of collagens) to explore the roles of epithelial-to-mesenchymal transition (EMT), the Wnt signaling pathway, and extracellular matrix (ECM) components in the progression and prognosis of NSCLC. The findings revealed that these markers were closely associated with tumor invasion and overall survival, highlighting their importance in NSCLC progression. Specifically, the study showed that WNT5A played a significant role in driving EMT and ECM remodeling, which were critical processes in tumor metastasis. Additionally, the study established correlations between these molecular markers and clinicopathological features, suggesting that they could serve as potential prognostic tools. The research enhanced our understanding of the molecular mechanisms driving NSCLC and indicated that these pathways and proteins might be viable targets for future therapeutic approaches Baldavira et al.

Novel biomarkers hold transformative potential in lung cancer management. They are central to the future of lung cancer care, playing a key role in everything from early detection and personalized treatment to ongoing disease monitoring. As research continues to advance, focusing on validating and implementing these novel biomarkers is crucial for driving personalized medicine in lung cancer. These efforts are essential for bringing the medical community closer to achieving more effective, individualized treatment strategies, ultimately improving patient outcomes and reducing the burden of lung cancer.
